# *Toxocara canis*- and *Toxocara cati*-Induced Neurotoxocarosis Is Associated with Comprehensive Brain Transcriptomic Alterations

**DOI:** 10.3390/microorganisms10010177

**Published:** 2022-01-14

**Authors:** Patrick Waindok, Elisabeth Janecek-Erfurth, Dimitri L. Lindenwald, Esther Wilk, Klaus Schughart, Robert Geffers, Christina Strube

**Affiliations:** 1 Institute for Parasitology, Centre for Infection Medicine, University of Veterinary Medicine Hannover, 30559 Hanover, Germany; patrick.waindok@freenet.de (P.W.); Elisabeth.Janecek@gmx.de (E.J.-E.); dimitril@gmx.net (D.L.L.); 2 Department of Infection Genetics, Helmholtz Centre for Infection Research, 38124 Braunschweig, Germany; Wilk@rochusmummert.com (E.W.); Klaus.Schughart@helmholtz-hzi.de (K.S.); 3 Department of Microbiology, Immunology and Biochemistry, University of Tennessee Health Science Centre, Memphis, TN 38163, USA; 4 Institute for Animal Breeding and Genetics, University of Veterinary Medicine Hannover, 30559 Hanover, Germany; 5 Research Group Genome Analytics, Helmholtz Centre for Infection Research, 38124 Braunschweig, Germany; robert.geffers@helmholtz-hzi.de

**Keywords:** neural larva migrans, toxocarosis, toxocariasis, gene transcription analysis, differential transcription, cholesterol biosynthesis, neurodegeneration, Alzheimer’s disease, microarray

## Abstract

*Toxocara canis* and *Toxocara cati* are globally occurring zoonotic roundworms of dogs and cats. Migration and persistence of *Toxocara* larvae in the central nervous system of paratenic hosts including humans may cause clinical signs of neurotoxocarosis (NT). As pathomechanisms of NT and host responses against *Toxocara* larvae are mostly unknown, whole-genome microarray transcription analysis was performed in cerebra and cerebella of experimentally infected C57Bl/6J mice as paratenic host model at days 14, 28, 70, 98, and 120 post-infection. Neuroinvasion of *T. cati* evoked 220 cerebral and 215 cerebellar differentially transcribed genes (DTGs), but no particular PANTHER (Protein ANalysis THrough Evolutionary Relationships) pathway was affected. In *T. canis*-infected mice, 1039 cerebral and 2073 cerebellar DTGs were identified. Statistically significant dysregulations occurred in various pathways, including cholesterol biosynthesis, apoptosis signaling, and the Slit/Robo mediated axon guidance as well as different pathways associated with the immune and defense response. Observed dysregulations of the cholesterol biosynthesis, as well as the Alzheimer disease-amyloid secretase pathway in conjunction with previous histopathological neurodegenerative findings, may promote the discussion of *T. canis* as a causative agent for dementia and/or Alzheimer’s disease. Furthermore, results contribute to a deeper understanding of the largely unknown pathogenesis and host-parasite interactions during NT, and may provide the basis for prospective investigations evaluating pathogenic mechanisms or designing novel diagnostic and therapeutic approaches.

## 1. Introduction

The dog and cat roundworm *Toxocara canis* and *Toxocara cati* are globally occurring intestinal parasites with canids and felids as definitive hosts. Due to the transpacental and/or lactogenic transmission to the offspring, they are of particular veterinary importance. Infections in dogs and cats have been associated with a variety of pathologies, induced either by the tracheal or somatic migration of larvae or adult nematodes in the gastrointestinal tract [1]. The global pooled prevalence of *Toxocara* infection in dogs is estimated at 11.1% and in cats at 17.0% [2,3], with significantly higher prevalences in young, rural, or stray animals. An estimated ≥100 million dogs and cats are infected and contaminate the environment with roundworm eggs [2,3]. Besides definitive hosts, a broad range of animals including humans can act as paratenic hosts after accidental ingestion of embryonated third-stage larvae (L3) containing eggs or L3 in tissues. 

Toxocarosis is one of the most frequently occurring zoonotic helminthoses worldwide [4], with an estimated global seroprevalence rate of 19% [5]. As in other paratenic hosts, human *Toxocara* infection is characterized by migration and persistence of L3 in paratenic host tissues. Clinical outcome and severity of toxocarosis depend on the intensity of infection, the duration and distribution of larval migration as well as occurring symptoms [6]. Accumulation and persistence of *Toxocara*-larvae in the central nervous system (CNS) may induce a syndrome called neurotoxocarosis (NT) [7,8]. However, the complex pathogenesis of NT results in a challenging diagnosis, wherefore the prevalence of NT is probably underestimated [9,10]. Common clinical findings are mostly non-specific, comprising encephalitis, myelitis, cerebral vasculitis, and eosinophilic meningoencephalitis, but NT has also been implicated to induce various neuropsychological disorders as well as neurological and cognitive deficits [7,8,11,12,13,14]. *Toxocara canis* is considered the causative agent of most human NT cases, wherefore the majority of experimental NT studies in rodents as model organisms have focused on this species [15]. Nevertheless, neuroinvasion of *T. cati* larvae also induces NT, but has only been marginally studied to date [15,16,17,18]. 

Most knowledge on NT is derived from only a small number of clinical cases or correlations of neurological disorders with *Toxocara*-seropositivity in humans [8] and a few studies only investigating molecular pathogenic mechanisms in laboratory animal models [15]. The progression of the disease in *T. canis*-infected mice is characterized by demyelination, focal malacia, mixed-cell infiltration as well as congestion, and neuronal necrosis [19,20]. At the molecular level, NT alters the transcription and expression patterns of different myelin marker genes [21]. Furthermore, neuroinvasion of *Toxocara* spp. impacts the complex molecular signaling network in infection and inflammation. For example, levels of anti-inflammatory immunoregulative molecules, including the cytokines interleukin IL-4 and IL-5 as well as regulatory lipid mediators such as neuroprotectin D1 are increased [22,23]. Furthermore, the ratio of anti-inflammatory 13- hydroxyoctadecadienoic acid (HODE) to pro-inflammatory 9-HODE is shifted in favor of 13-HODE [22]. Janecek et al. [24] identified a plethora of differentially transcribed genes (DTGs) in brains of *T. canis*- and *T. cati*-infected C57BL/6J mice as paratenic host model at day 42 post-infection (pi), where DTGs were mainly associated with the host´s immune response, especially the defense/inflammatory response and lipid/cholesterol biosynthetic processes. However, many questions regarding pathogenic mechanisms and immunogenic reactions during NT have not been clarified yet. Therefore, the aim of this study was to perform an extensive characterization of transcriptional changes during the course of neuroinfection. Such identification of key regulatory mechanisms is necessary for an enhanced understanding of the largely unknown pathogenesis and mechanisms of host-parasite interactions during *T. canis*- and *T. cati*-induced NT.

## 2. Materials and Methods

### 2.1. Experimental Infection

Experimental infection was performed as previously described [24]. Briefly, eggs of *Toxocara canis* (field isolate HannoverTcanis2008) and *Toxocara cati* (field isolate HannoverTcati2010) were collected from feces of experimentally infected dogs and cats, respectively, maintained at the Institute for Parasitology, University of Veterinary Medicine Hannover, Germany. Eggs were purified from the feces by a combined sedimentation-flotation technique and incubated at 25 °C in tap water for about four weeks to allow for embryonation. Afterward, eggs were stored at 4 °C until infection. 

A total of 90 5-week-old male and female C57BL/6JRccHsd were purchased (Harlan Laboratories, Horst, the Netherlands) and acclimatized for a period of one week. Mice were housed in Makrolon cages in a 12/12 h dark/light cycle, receiving a standard rodent diet (Altromin 1324, Altromin, Lage, Germany) and water ad libitum. At the age of 6 weeks, mice were randomly allocated to the control and infection groups of 30 animals each. Animals of the *T. canis*- and *T. cati*-group were orally infected with 2000 embryonated *T. canis* or *T. cati* eggs, respectively, in a total volume of 0.5 mL tap water. The control group received the same volume of tap water only. On days 7, 14, 28, 70, 98, and 120 pi, every five mice of the *T. canis*- and *T. cati*-infection group, as well as the control group, were euthanized. Brains were carefully removed, divided into the right and left hemispheres, and hemispheres were separated into cerebrum and cerebellum. The cerebra and cerebella of the right hemispheres of were individually stored in RNA*later™* (Qiagen, Hilden, Germany) according to the manufacturer’s instructions and frozen at −80 °C until further processing. 

### 2.2. RNA Isolation and Processing for Microarray Experiments

RNA was isolated from approx. 100 mg of each cerebrum and cerebellum and processed for microarray analysis as previously described [24] using the RNeasy^®^ Lipid Tissue Mini kit (Qiagen, Hilden, Germany), including a DNase digestion step according to the manufacturer’s instructions. The quality and integrity of the total RNA (28S/18S rRNA ratio and RNA integrity number, respectively) were controlled using the Agilent Technologies 2100 Bioanalyzer (Agilent Technologies, Waldbronn, Germany). For three samples per group, time point, and brain region were used for microarray experiments. Microarray analysis was conducted as described by Janecek et al. [24] by labeling 100 ng of sample RNA with Cy3 according to the one-color Quick Amp Low Input Labeling protocol (Agilent Technologies, Waldbronn, Germany). Labeled cRNA was hybridized to Agilent’s 4 × 44 k Mouse V2 (Agilent Technologies, Waldbronn, Germany), Design ID: 026655 for 17 h at 65 °C and scanned as described by Pommerenke et al. [25].

### 2.3. Low-Level Analyses

Initial processing of microarray data included background subtraction as well as data normalization using R (v.3.1.2, R Foundation for Statistical Computing, Vienna, Austria [26]) with the packages “limma” (Linear Models for Microarray and RNA-Seq Data) and “sva” (Surrogate Variable Analysis). The supervised sva (ssva) approach included in the “sva” package was conducted to adjust batch effects, with integrated control probes selected as a reference. To determine inter- and intra-group-specific variations and to confirm biological reproducibility, principal component analysis (PCA) was applied using the R package “affycoretools”.

### 2.4. Identification of Differentially Transcribed Microarray Probes and Corresponding Genes

Differentially transcribed microarray probes (DTPs) were determined by SAM (Significance Analysis for Microarrays; R package “samr”), limma (Linear Models for Microarray and RNA-Seq Data; R package “limma”), and maSigPro (Microarray Significant Gene Expression Profiles; R package “maSigPro”). Selected criteria for each package were set as follows: SAM (del = 1.079 for cerebrum and del = 1.796 for cerebellum to keep FDR computed by delta.table <10%, min. fold change = 0), limma (*p* = 0.05, adjustment by Benjamini-Hochberg, with weights by group), and maSigPro (step.method “backward”, alfa = 0.05, rsq = 0.65, vars = “groups”). To reduce package-specific bias, solely probes and corresponding genes identified as differentially transcribed by all three methods were considered for the investigation. The intersection of package-derived DTPs was compared and visualized with Venny v.2.1 [27]. DTPs were assigned to corresponding differentially transcribed genes (DTGs) using R packages “annotate” and “MmAgilentDesign026655.db” v.3.2.2.

### 2.5. Pathway Analyses

Association of identified DTGs with regulatory and metabolic pathways was conducted with the statistical overrepresentation test with Fisher’s exact test and adjusted by the false discovery rate (FDR) correction provided by the Protein ANalysis THrough Evolutionary Relationships (PANTHER) database v.16.0 [28], using the PANTHER Pathways as annotation dataset for *Mus musculus* [29]. Pathways with an FDR-adjusted *p*-value ≤ 0.05 were considered statistically significantly enriched.

## 3. Results

### 3.1. Clinical Assessment

Mice infected with either *T. canis* or *T. cati* showed reduced body weights compared to uninfected control mice. As previously observed by Janecek et al. [30], infected mice were exposed to varying degrees of clinical symptoms and neurobehavioral alterations. Alterations in both infection groups mainly started around day 6 pi with progressively increasing severity during the course of infection, although *T. cati*-induced neurotoxocarosis was generally characterized by less severe clinical signs and neurobehavioral alterations.

### 3.2. Microarray Data

The microarray data set is available at the NCBI’s Gene Expression Omnibus (GEO) database under the accession number GSE190123.

### 3.3. Principal Component Analysis

**Figure 1:**
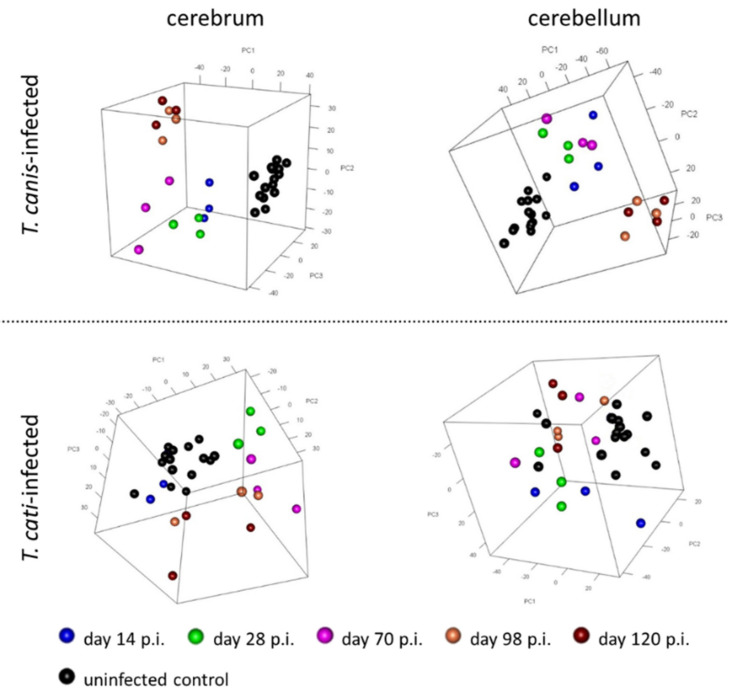
Principal component analysis of transcriptional changes in cerebra and cerebella of *T. canis*- and *T. cati*-infected C57Bl/6J mice at different time points post-infection. To provide a better overview, all respective control mice are shown in black.

Overall, PCA indicated differential gene transcriptions between *Toxocara*-infected and uninfected control mice as well as both brain areas. However, day 7 pi samples of *T. canis*- and *T. cati*-infected mice showed considerable transcriptional divergences between each other and were therefore excluded from further analyses. As of day 14 pi, group data trended in a similar manner, especially in the *T. canis* infected mice, where PCA plots showed different clustering between cerebrum and cerebellum as well as a clear distinction to the uninfected control mice. Furthermore, samples from day 98 and day 120 pi of both brain parts clustered separately from the earlier study days (Figure 1). The *T. cati*-infected mice also showed distinct transcriptomic differences between the cerebrum and cerebellum, but sample clustering of the different time points was less pronounced (Figure 1). This was particularly true for the cerebellum, where no clear transcriptional differences could be observed compared to the uninfected control mice.

### 3.4. Differentially Transcribed Microarray Probes and Corresponding Genes in Brains off T. canis-Infected Mice

**Figure 2:**
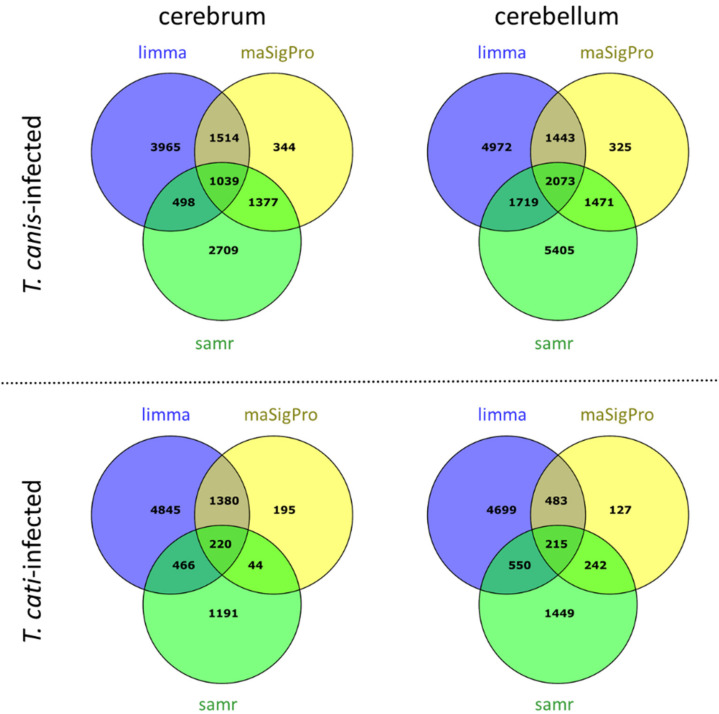
Differentially transcribed genes identified in cerebra and cerebella of *T. canis*- and *T. cati*-infected mice over the course of infection.

Transcriptional data analyses using R packages SAM, limma, and maSigPro revealed 1239 differentially transcribed microarray probes (DTPs) in the cerebrum during the course of *T. canis* infection. As 132 DTPs could not be annotated by the R package “annotate”, and 68 probes were replicates for different genes, a total of 1039 differentially transcribed genes (DTGs) could be identified (Figure 2). Of these, 477 DTGs were upregulated and 289 were downregulated at each time point of the study. The remaining 273 DTGs did not show a consecutive trend and were either up- or downregulated at different time points of the analysis. 

In the cerebellum, the infection with *T. canis* resulted in 2544 DTPs during the course of infection, corresponding to 2073 DTGs, as 304 probes could not be annotated and 167 probes were replicates of different genes. Of the total number of DTGs, 884 DTGs were upregulated and 599 were downregulated at each time point of the analysis. A varying dysregulation over the course of infection was observed for 590 DTGs.

### 3.5. Differentially Transcribed Microarray Probes and Corresponding Genes in Brains of T. cati-Infected Mice

During the course of *T. cati*-induced brain infection, a total of 274 microarray probes were differentially transcribed in the cerebrum, resulting in 220 DTGs (Figure 2), as 48 DTPs could not be annotated and six DTPs were replicates of different genes. A consistent upregulation was observed for six DTGs, while nine DTGs were downregulated at each time point of the analysis, and 205 DTGs varied in their transcriptional regulation during the course of infection. 

Cerebellar neuroinvasion of *T. cati* was characterized by 263 DTPs, corresponding to 215 identified DTGs, as 35 DTPs could be annotated and 13 DTPs were replicates. The infection evoked the upregulation of 127 DTGs and downregulation of DTGs throughout the course of infection, while the regulation of the remaining 69 DTGs did not show a distinct trend.

### 3.6. DTG Overlap in Cerebra and Cerebella of T. canis- and T. cati-Infected Mice 

**Figure 3:**
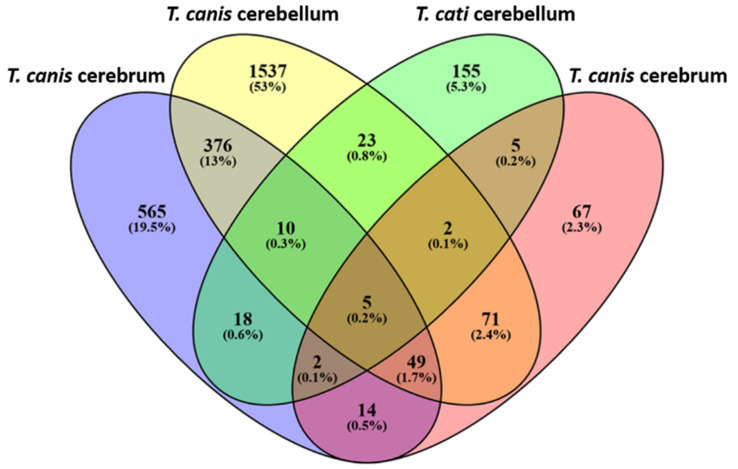
Venn diagram showing number and percentage of specific and overlapping differentially transcribed genes in *T. canis*- and *T. cati*-infected cerebra and cerebella.

**Figure 4:**
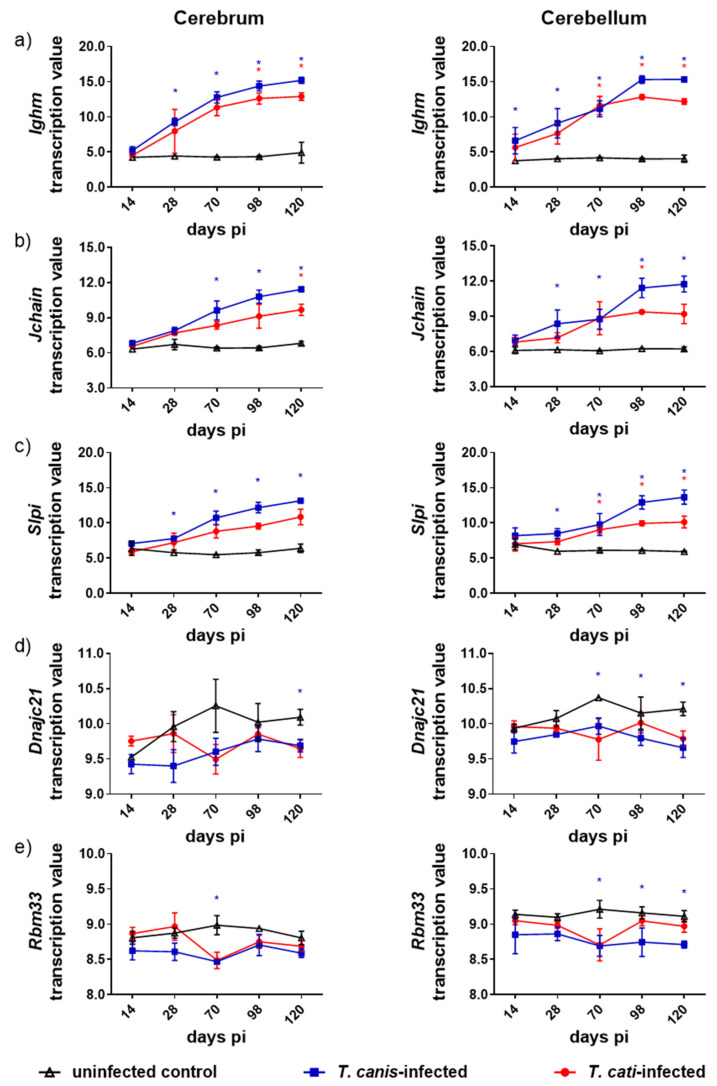
Transcriptional values of the five genes (**a**) *Ighm*, (**b**) *Jchain*, (**c**) *Slpi*, (**d**) *Dnajc21*, and (**e**) *Rbm33* differentially transcribed in the cerebra and cerebella of both *Toxocara. canis*- and *T. cati*-mice at different days post infection (pi). Asterisks indicate statistically significant differences (*p* ≤ 0.05) between infected and uninfected mice according to the color scheme.

Comparative DTG analysis showed that 2324 genes were solely dysregulated in either the cerebrum or the cerebellum of *T. canis*- or *T. cati*-infected mice, respectively (Figure 3). Regarding DTG overlap, 507 genes were dysregulated in two overlapping subsets, while 63 genes were affected in three overlapping subsets. Only five genes were dysregulated in the cerebrum and cerebellum of *T. canis*- as well as *T. cati*-infected mice. Of these five genes, the transcription values of *Ighm*, *Jchain,* and *Slpi* increased progressively during the course of infection in both brain areas of *T. canis* as well as *T. cati*-infected mice (*p* = 0.0294) (Figure 4). Transcription of *Dnajc21* and *Rbm33* was rather downregulated, strikingly with statistically significant decreased values during the chronic phase (as of day 70 pi) of cerebellar *T. canis* infection (*p* = 0.0177).

### 3.7. PANTHER Pathway Analysis of DTGs

An overview of statistically significantly dysregulated PANTHER pathways in cerebra and cerebella of *T. canis*-infected mice is given in Table 1. Of the 1039 DTGs in the cerebrum of *T. canis*-infected mice, 915 DTGs mapped uniquely, 21 had multiple mapping information, and 103 could not be mapped in PANTHER pathways. The broad majority of mapped DTGs (790) were marked as “unclassified” and did not cluster in specific pathways. A total of 125 DTGs were mapped in 9 statistically significantly dysregulated pathways. The infection affected the cholesterol biosynthesis (fold enrichment = 10.76, adj. *p* = 0.003), including six predominately downregulated genes. In contrast, transcription of the 13 affected DTGs in the interleukin signaling pathway (fold enrichment = 3.44, *p* = 0.005) was mostly upregulated during the course of infection. No distinct trend was observed for the remaining pathways, where genes were either up- or downregulated during the course of infection.

In the cerebella of *T. canis*-infected mice, 249 out of the 2073 DTGs remained unmapped by PANTHER pathways analysis, while 1824 could be mapped. Of these, 1643 DTGs mapped uniquely and 181 had multiple mapping information. While 1542 DTGs were marked as “unclassified”, 101 DTGs were dysregulated in three statistically significantly affected pathways; apoptosis signaling, chemokine, and cytokine signaling as well as integrin signaling (Table 1). Within these pathways, the different DTGs did not show a distinct trend, but were either up- or downregulated. 

The infection with *T. cati* resulted in 220 DTGs in the cerebrum, of which 199 DTGs were mapped, while 21 DTGs could not be allocated in specific PANTHER pathways. In the cerebellum, 195 of the 215 DTGs could be mapped and 20 DTGs did not cluster. However, *T. cati* infection did not result in statistically significant transcriptional pathway alterations in either the cerebrum or the cerebellum.

### 3.8. Enriched PANTHER Pathways at Different Study Days

The number of DTGs and statistically significantly affected pathways for each study day during the course of infection are provided in Table 2. The number of downregulated genes in the cerebra of *T. canis*-infected mice varied from 418 (day 120 pi) to 505 (day 70 pi). The cholesterol biosynthesis was the only pathway with statistically significantly decreased transcription rates at each time point of the study. In the advanced chronic phase of infection, i.e., days 98 and 120 pi, the heterotrimeric G-protein signaling pathway was additionally downregulated. Further pathways like the gonadotropin-releasing hormone receptor pathway or the Alzheimer disease-amyloid secretase pathway were statistically significantly affected by downregulated DTGs at day 120 pi.

The number of upregulated genes in the cerebra of *T. canis*-infected mice varied between 534 DTGs at day 70 pi and 621 DTGs at day 120 pi. Gene transcription of DTGs in the apoptosis-, JAK/STAT-, Interleukin-, CCKR- and chemokine and cytokine-mediated signaling pathways as well as the B- and T-cell activation was mostly upregulated throughout the whole study period. Additionally, the Ras pathway was significantly affected at days 14, 28, 70, and 98 pi, and the Slit/Robo mediated axon guidance pathway at days 14, 70, and 120 pi by upregulated DTGs. In the FAS signaling pathway, DTGs were significantly upregulated in the subacute phase (days 14 and 28 pi) and the beginning of the chronic phase (day 70 pi) of infection. 

In the cerebella of *T. canis*-infected mice, the number of downregulated DTGs varied between 934 DTGs at day 28 pi and 1091 DTGs at day 98 pi, but no particular pathway was overrepresented in the PANTHER pathway analysis. Upregulation of genes varied between 1017 DTGs at day 70 pi and 1139 DTGs at day 28 pi. During the entire study period, the Toll receptor-, Apoptosis-, Interleukin- and chemokine and cytokine-mediated signaling, as well as the B-cell activation pathway, included statistically significant numbers of upregulated DTGs. The T-cell activation and the Cadherin signaling pathway were significantly affected in the subacute phase of infection at day 28 as well as the latest time point of the analysis, day 120 pi. The Plasminogen activating cascade and the JAK/STAT signaling pathway were significantly altered in the chronic phase-only i.e., at days 70, 98, and 120 pi and days 98 and 120 pi. 

During the course of infection with *T. cati*, the number of downregulated genes varied between 52 DTGs and 78 DTGs at days 70 and 120 pi in the cerebra and between 52 DTGs and 55 DTGs at days 14 and 120 pi in the cerebella. Upregulation of DTGs in the cerebra of *T. cati*-infected mice varied between 142 DTGs at day 120 pi and 168 DTGs at day 70 pi. In the cerebella, the range of upregulated DTGs was 160 DTGs at day 120 pi and 163 DTGs at day 14 pi. However, although the infection with *T. cati* resulted in differentially transcribed genes in both brain parts, no particular pathway was statistically significantly affected.

## 4. Discussion

The progressive migration and accumulation of larvae in somatic and cerebral tissues classifies NT into an acute (approx. until day 14 pi), subacute (approx. until day 28 pi), and subsequent chronic phase of infection [15]. The course of infection is characterized by the onset of neurobehavioral changes and inflammatory reactions in the brain of infected paratenic hosts in the acute and subacute phase, as well as progressively increasing behavioral and pathological effects during the chronic phase [20,31,32]. The CNS manifestation in humans is associated with different neuropsychological disorders and cognitive deficits [7,8,11,12,13,14]. Furthermore, murine NT, used as a model for human NT, is characterized by neurological and motor dysfunctions, reduced levels of aggressive behavior and anxiety as well as impairments of learning and memory capacity [30,32,33]. Larval migration into the cerebral tissues causes parenchymal damage with macroscopically visible hemorrhagic lesions, while the further course of infection results in neurodegenerative processes [20,21,31,34]. Although larvae of *T. canis* are regarded as the common causative agent of human NT, *T. cati* is also capable of inducing NT [15,16,17,18]. Neurological disorders and histopathological alterations are prominent in both infections, but the progression of the disease is pronounced in *T. canis*-induced infections [15,19,21,31,35]. These differences in the severity of NT are reflected by alterations in transcriptomic profiles of both infection groups in the present study. The combined SAM, limma and maSigPro microarray analysis resulted in a considerably higher number of DTGs in *T. canis*-infected mice compared to *T. cati*-infected mice, with 1239 and 2544 DTGs in the cerebrum and the cerebellum of *T. canis*-infected mice. In contrast, only 274 and 263 DTGs were identified in the cerebrum and cerebellum upon *T. cati* infection. These differences are further evident at the particular study days, where the number of DTGs was always higher in both brain parts of the *T. canis*-infected mice than in the *T. cati*-infected mice. Nevertheless, five genes were dysregulated in the cerebrum as well as the cerebellum by both pathogens. Interestingly, three of them—*Ighm*, *Jchain*, and *Slpi*—showed the same pattern in terms of a progressive increase during the course of infection in both brain parts and infection groups. These genes are involved in general immune reactions, as *Ighm* and *Jchain* encode parts of immunoglobulins and *Slpi* encodes a secreted inhibitor that protects epithelial tissues [36,37,38].

Individual DTGs were categorized by PANTHER classification, revealing the cholesterol biosynthesis pathway as one of the most affected pathways in the cerebrum of *T. canis*-infected mice. Cholesterol is an important structural component for cellular membranes, a precursor of steroid hormones, and mandatory in myelin formation, enabling the saltatory conduction of action potentials by discontinuous insulation of neurons [39]. Therefore, cholesterol is necessary for the maintenance of the synaptic function of neurons, and depletion of cholesterol with synaptic loss is frequently observed in neurodegenerative diseases [40]. Interestingly, transcription of genes in this pathway was predominantly decreased during the whole course of infection. Compared to the uninfected control group, DTGs of other pathways were downregulated only in the chronic phase at days 98 and 120 pi or upregulated during the study period. Transcription rates of genes involved in the cholesterol pathway also decrease during neuroinfection with *Neospora caninum* as well as in scrapie-inoculated mice, resulting in the exacerbation of neuronal damage, neurodegeneration, impaired regeneration of damaged neuronal tissues, and the disruption of neurotransmission [41,42]. 

Along with decreased transcriptions in the cholesterol biosynthesis pathway, the infection with *T. canis* was characterized by elevated mRNA levels of genes associated with the apoptosis signaling pathway. Dysregulation of apoptotic processes has been linked to numerous pathologies, such as chronic inflammation and neurodegenerative diseases like Alzheimer’s and Parkinson’s diseases [43]. Infections with *T. canis* are characterized by progressive neuronal demyelination with a focal accumulation of gitter cells, displaying myelinophagia as well as spheroids and cholesterol crystals, indicating axonal damage [20,31,44]. The significant dysregulation of cholesterol biosynthesis, as well as the upregulation of the apoptosis signaling pathway, may promote the pathological outcome of NT, but further studies are required to strengthen this hypothesis. 

Upon *T. cati* infection, however, neither the cholesterol biosynthesis nor the apoptosis signaling pathway was significantly affected compared to the uninfected control mice. Assuming a predominant role of these pathways in the clinical outcome of NT, the less severe progression of the *T. cati*-induced disease may be reflected by the transcriptional outcome of this study. 

Further dysregulated pathways during *T. canis* infection participate in neuroinflammatory processes and the cerebral immune response, e.g., the B- and T-cell activation or the chemokine and cytokine-mediated signaling pathways. Host- or parasite-induced immunoregulatory processes may contribute to the pathogenesis of NT, but detailed data on the involvement of signaling molecules during cerebral *Toxocara* infections are scarce [15]. Important molecules involved in the complex molecular signaling network in infection and inflammation are cytokines and chemokines as well as polyunsaturated fatty acid-derived bioactive regulatory lipids such as oxylipins. Neuroinvasion of larvae did not have a promoting effect on metabolite concentrations of pro-inflammatory mediators like TNF-α, IFN-γ, GM-CSF, and IL-6 [22], but led to a prominent increase in anti-inflammatory immunoregulative molecules in infected brains, including cytokines IL-4, IL-5, the chemokine eotaxin, and 12/15-LOX-products, e.g. 13-HODE or neuroprotectin D1 [22,23]. The increase in anti-inflammatory cytokines and chemokines is also reflected at the microarray level, as pathways like the interleukin signaling pathway or the inflammation mediated by chemokine and cytokine signaling pathway were affected by brain infection with *T. canis*. However, an encompassing trend in the transcriptional regulation of the neuroinflammatory pathways was not observed as genes were either up- or downregulated during the course of infection. 

Although infection with *T. canis* resulted in elevated numbers of DTGs in the cerebellum, only three pathways were significantly dysregulated during the course of infection. Along with apoptosis signaling and inflammation mediated by chemokine and cytokine signaling, the integrin signaling pathway was influenced by neuroinvasive *T. canis* larvae. Integrins, as transmembrane adhesion receptors, mediate cell-cell and cell-extracellular matrix adhesion, influence various signal transduction cascades in control of cell survival, proliferation, differentiation, and organ development [45]. Furthermore, integrin-mediated adhesive interactions are essential in regulating different selective cell responses, such as transmigration into the inflammatory site, cytokine secretion, and production of reactive oxygen intermediation of polymorphonuclear leukocytes (PMNs) as well as monocyte/macrophages [46]. These interactions may participate in the NT-characteristic infiltration of eosinophilic granulocytes in cerebral tissues [20,47]. Different genes of the integrin signaling pathway were downregulated in our analysis, while transcriptional levels of *Itgax* (CD11c), encoding the integrin subunit alpha X, were elevated in cerebra of *T. canis*-infected mice. CD11c seems to play a major role in inducing the Th2 response, as gene depletion resulted in an impaired Th2 cytokine profile to a stimulus with *Schistosoma mansoni* [48]. Interestingly, the cerebral immune response in *T. canis*-induced NT evokes a Th2-polarised immune reaction [23].

Despite the progressive course of NT, most pathways identified as overrepresented in the day-wise PANTHER classification were dysregulated in both the cerebra and cerebella of *T. canis*-infected mice at the most examined time points. Pathways predominately associated with the immune response, but also the apoptosis signaling pathway showed continuously increased transcription rates. Constantly decreased transcriptional levels at each study day could be detected in the cerebra of *T. canis*-infected mice only for genes associated with cholesterol biosynthesis. Additionally, the number of dysregulated pathways with decreased transcriptional levels increased in the advanced chronic phase of infection. Of these, the Alzheimer disease-amyloid secretase pathway affected at day 120 pi is of particular interest in terms of neurodegeneration as a consequence of *Toxocara* neuroinvasion. Furthermore, *T. canis* is discussed as a potential causative agent for Alzheimer’s disease (AD) [8,20,24]. Axonal damage upon NT is indicated by accumulated β-amyloid precursor protein (β-APP), which, in combination with its derivatives like the amyloid β protein (Aβ) plays a pivotal role in AD [49]. During the analysis, DTGs included in the Alzheimer disease-amyloid secretase pathway were *Prkcd* (protein kinase C δ [PKC-δ]) and *Mapk10* (mitogen-activated protein kinase 10 [Mapk10]). Stimulation of microglia with Aβ increased the kinase activity of PKC-δ [50], and PKC-δ expression is elevated in human AD, correlating with beta-site APP cleaving enzyme 1 (BACE1) expression in BACE1-mediated β-APP processing as well as Aβ production [51]. Furthermore, patients with AD had significantly increased levels of Mapk10, associated with Aβ in senile plaques [52].

A further dysregulated pathway with decreased transcriptional levels compared to the uninfected control mice was the gonadotropin-releasing hormone (GnRH) receptor pathway. The peptide GnRH is primarily known to participate in the regulation of reproduction via the hypothalamic-pituitary-gonadal axis, but may also serve as a neurotrophic factor and modulator of hippocampal synaptic activities, impacting central nervous system physiology as well as pathophysiology [53,54,55,56]. Reduced levels of GnRH may contribute to the development of AD pathology, as the treatment with GnRH lowered hippocampal plaque load, reduced the Aβ concentration in brains of C57BL/6 mice, and prevented AD-related cognitive dysfunctions [57,58]. Additionally, inflammatory processes in the hypothalamus of aged mice result in downregulation of GnRH expression [59]. Cognitive function deficits are also associated with deficiency of the growth hormone (GH), another hypothalamic hormone-induced, for example, by traumatic brain injuries [60,61,62]. Interestingly, memory impairment, as well as cognitive dysfunction, have also been described as a consequence of NT in humans [8,12,14].

The infection with *T. canis* resulted in a higher number of DTGs in the cerebellum than in the cerebrum, but PANTHER Classification analysis resulted in a higher number of affected pathways in the cerebrum, which may reflect the described species-specific brain part tropism of *T. canis* larvae in paratenic hosts [31]. In contrast, *T. cati* larvae rather migrate to the cerebellum than to the cerebrum, but larvae mainly accumulate in muscle tissue [31]. Consequently, fewer DTGs were observed in the brain of *T. cati*-infected mice and no particular pathway was overrepresented during the PANTHER Classification analysis. This mirrors previous observations in *T. cati*-infected C57BL/6J mice, which showed delayed onset and less severe progression of neurobehavioral alterations as well as less severe histopathological alterations compared to *T. canis*-infected mice [15,21,30]. 

Finally, an important point to mention is that NT is characterized by focally distributed lesions, so healthy brain tissue was most likely overrepresented in the transcriptional analysis compared to damaged tissue. This may have masked local effects induced by migrating or persisting *T. cati* larvae, and may have reduced observed effects of *T. canis* larvae on both brain parts. 

## 5. Conclusions

*T. canis*- and *T. cati*-induced neurotoxocarosis in the paratenic mouse model was associated with the comprehensive transcriptional alteration. Nevertheless, the known differences between the pathogens were reflected by substantially higher numbers of DTGs in brain tissues of *T. canis*- compared to *T. cati*-infected mice. The prominent pathogenesis of *T. canis*-induced NT was also evident in the affected pathways. While the PANTHER analysis of DTGs in brains of *T. cati*-infected mice did not result in the overrepresentation of specific pathways, neuroinvasion of *T. canis* larvae evoke dysregulation of various pathways associated with the immune response. Furthermore, cerebral transcription of genes associated with cholesterol biosynthesis was constantly decreased during the course of infection, suggesting a fundamental impact of neuroinvasive *T. canis* larvae on cholesterol homeostasis. Dysregulation of the cholesterol biosynthesis pathway may provoke neurodegeneration, a typical hallmark of NT. Thus, axonal damage in conjunction with the dysregulated Alzheimer disease-amyloid secretase pathway promotes the discussion of *T. canis* as a causative agent for Alzheimer’s disease. Further research investigating these and other topics is required to characterize the still largely unknown pathogenesis of *T. canis*- and *T. cati*-induced NT and to develop new diagnostic and therapeutic approaches.

## Figures and Tables

**Table 1:** Pathways affected by differentially transcribed genes (DTGs) in *T. canis*-infected cerebra and cerebella. Note that no pathways were statistically significantly overrepresented during *T. cati*-infection.

	Enriched Biological Modules (Panther Pathway Accession Number)	DTGs	Fold-Enrichment	Adj. *p*-Value (FDR)
Cerebrum	Cholesterol biosynthesis (P00014)	6	10.76	0.003
	Axon guidance mediated by Slit/Robo (P00008)	7	6.80	0.005
	B cell activation (P00010)	13	4.33	0.001
	T cell activation (P00053)	14	3.63	0.003
	Apoptosis signaling pathway (P00006)	18	3.56	0.001
	Interleukin signaling pathway (P00036)	13	3.44	0.005
	Ras Pathway (P04393)	10	3.33	0.030
	CCKR signaling map (P06959)	21	3.08	0.001
	Inflammation mediated by chemokine and cytokine signaling pathway (P00031)	23	2.08	0.034
	Unclassified	790	0.93	<0.001
Cerebellum	Apoptosis signaling pathway (P00006)	25	2.54	0.006
	Inflammation mediated by chemokine and cytokine signaling pathway (P00031)	44	2.04	0.004
	Integrin signaling pathway (P00034)	32	2.02	0.023
	Unclassified	1542	0.94	<0.001

**Table 2:** Number of differentially transcribed genes (DTGs) in *Toxocara canis*- and *T. cati*-infected cerebra and cerebella and enriched biological pathways with an adjusted *p*-value ≤ 0.05 for each study day.

	Study Day	Regulation	Number of DTGs	Enriched Biological Modules (PANTHER Pathway Accession Number)
*T. canis*				
cerebrum	14 pi	up	605	Apoptosis signaling pathway (P00006), Axon guidance mediated by Slit/Robo (P00008), B-cell activation (P00010), CCKR signaling map (P06959), FAS signaling pathway (P00020), Inflammation mediated by chemokine and cytokine signaling pathway (P00031), Interleukin signaling pathway (P00036), JAK/STAT signaling pathway (P00038), Ras Pathway (P04393), T-cell activation (P00053)
		down	434	Cholesterol biosynthesis (P00014)
	28 pi	up	539	Apoptosis signaling pathway (P00006), B-cell activation (P00010), CCKR signaling map (P06959), FAS signaling pathway (P00020), Inflammation mediated by chemokine and cytokine signaling pathway (P00031), Interleukin signaling pathway (P00036), JAK/STAT signaling pathway (P00038), Ras Pathway (P04393), T-cell activation (P00053)
		down	500	Cholesterol biosynthesis (P00014)
	70 pi	up	534	Apoptosis signaling pathway (P00006), Axon guidance mediated by Slit/Robo (P00008), B-cell activation (P00010), CCKR signaling map (P06959), FAS signaling pathway (P00020), Inflammation mediated by chemokine and cytokine signaling pathway (P00031), Interleukin signaling pathway (P00036), JAK/STAT signaling pathway (P00038), Ras Pathway (P04393), T-cell activation (P00053)
		down	505	Cholesterol biosynthesis (P00014)
	98 pi	up	553	Apoptosis signaling pathway (P00006), B-cell activation (P00010), CCKR signaling map (P06959), FAS signaling pathway (P00020), Inflammation mediated by chemokine and cytokine signaling pathway (P00031), Interleukin signaling pathway (P00036), JAK/STAT signaling pathway (P00038), Ras Pathway (P04393), T-cell activation (P00053)
		down	486	Cholesterol biosynthesis (P00014), Heterotrimeric G-protein signaling pathway-Gq alpha and Go alpha mediated pathway (P00026)
	120 pi	up	621	Apoptosis signaling pathway (P00006), Axon guidance mediated by Slit/Robo (P00008), B-cell activation (P00010), CCKR signaling map (P06959), Inflammation mediated by chemokine and cytokine signaling pathway (P00031), Interleukin signaling pathway (P00036), JAK/STAT signaling pathway (P00038), T-cell activation (P00053)
		down	418	5HT2 type receptor-mediated signaling pathway (P04374), Alzheimer disease-amyloid secretase pathway (P00003), Cholesterol biosynthesis (P00014), Gonadotropin-releasing hormone receptor pathway (P06664), Heterotrimeric G-protein signaling pathway-Gq alpha and Go alpha mediated pathway (P00026)
cerebellum	14 pi	up	1106	Apoptosis signaling pathway (P00006), B-cell activation (P00010), Inflammation mediated by chemokine and cytokine signaling pathway (P00031), Interleukin signaling pathway (P00036), Toll receptor signaling pathway (P00054)
		down	967	n/a
	28 pi	up	1139	Apoptosis signaling pathway (P00006), B-cell activation (P00010), Inflammation mediated by chemokine and cytokine signaling pathway (P00031), Interleukin signaling pathway (P00036), T-cell activation (P00053), Toll receptor signaling pathway (P00054)
		down	934	n/a
	70 pi	up	1017	Apoptosis signaling pathway (P00006), B-cell activation (P00010), Inflammation mediated by chemokine and cytokine signaling pathway (P00031), Interleukin signaling pathway (P00036), Plasminogen activating cascade (P00050), Toll receptor signaling pathway (P00054)
		down	1056	n/a
	98 pi	up	1091	Apoptosis signaling pathway (P00006), B-cell activation (P00010), Inflammation mediated by chemokine and cytokine signaling pathway (P00031), Interleukin signaling pathway (P00036), JAK/STAT signaling pathway (P00038), Plasminogen activating cascade (P00050), Toll receptor signaling pathway (P00054)
		down	982	n/a
	120 pi	up	1084	Apoptosis signaling pathway (P00006), B-cell activation (P00010), Cadherin signaling pathway (P00012), Inflammation mediated by chemokine and cytokine signaling pathway (P00031), Interleukin signaling pathway (P00036), JAK/STAT signaling pathway (P00038), Plasminogen activating cascade (P00050), Toll receptor signaling pathway (P00054)
		down	989	n/a
*T. cati*				
cerebrum	14 pi	up	150	n/a
		down	70	n/a
	28 pi	up	165	n/a
		down	55	n/a
	70 pi	up	168	n/a
		down	52	n/a
	98 pi	up	158	n/a
		down	62	n/a
	120 pi	up	142	n/a
		down	78	n/a
cerebellum	14 pi	up	163	n/a
		down	52	n/a
	28 pi	up	161	n/a
		down	54	n/a
	70 pi	up	161	n/a
		down	54	n/a
	98 pi	up	161	n/a
		down	54	n/a
	120 pi	up	160	n/a
		down	55	n/a

n/a: no significantly enriched biological modules.

## Data Availability

The microarray data set is available at the Gene Expression Omnibus (GEO) database of the National Center for Biotechnology Information (NCBI) under the accession number GSE190123.
